# Hesperidin and Chlorogenic Acid Synergistically Inhibit the Growth of Breast Cancer Cells via Estrogen Receptor/Mitochondrial Pathway

**DOI:** 10.3390/life11090950

**Published:** 2021-09-10

**Authors:** Pang-Hung Hsu, Wei-Hsuan Chen, Chen Juan-Lu, Shu-Chen Hsieh, Shih-Chao Lin, Ru-Tsun Mai, Shiow-Yi Chen

**Affiliations:** 1 Department of Bioscience and Biotechnology, College of Life Sciences, National Taiwan Ocean University, No. 2, Beining Rd, Zhongzheng District, Keelung City 202, Taiwan; phsu@ntou.edu.tw (P.-H.H.); 1083B008@mail.ntou.edu.tw (W.-H.C.); 2 Center of Excellence for the Oceans, National Taiwan Ocean University, No. 2, Beining Rd, Zhongzheng District, Keelung City 202, Taiwan; 3 Institute of Biochemistry and Molecular Biology, National Yang Ming Chiao Tung University, No. 155, Section 2, Linong St, Beitou District, Taipei City 112, Taiwan; 4 Institute of Food Science and Technology, National Taiwan University, No. 1, Roosevelt Rd, Da’an District, Taipei City 10617, Taiwan; d04641005@ntu.edu.tw (C.J.); schsieh@ntu.edu.tw (S.-C.H.); 5 Bachelor Degree Program in Marine Biotechnology, College of Life Sciences, National Taiwan Ocean University, No. 2, Beining Rd, Zhongzheng District, Keelung City 202, Taiwan; sclin@mail.ntou.edu.tw; 6 Department of Biological Science & Technology, National Yang Ming Chiao Tung University, No. 75, Boai Street, Hsinchu 300, Taiwan; mairt@nctu.edu.tw

**Keywords:** breast cancer, synergistic effect, hesperidin, chlorogenic acid, estrogen receptor

## Abstract

Breast cancer is the most common cancer in women worldwide. Hesperidin (Hes) and chlorogenic acid (CA) are traditional medicinal molecules that abundantly exist in natural plants or foods. These compounds have been shown to prevent and suppress various cancers and therefore can be utilized as adjunctive therapies to aid cancer treatment. Here, 3-(4,5-Dimethylthiazol-2-yl)-2,5-diphenyltetrazolium bromide (MTT) assays show a greater synergistic inhibitory effect on the growth of breast cancer cells, MCF-7, but not normal breast cells, MCF-10A, than hesperidin or chlorogenic acid alone. We present the possible molecular signaling pathways in MCF-7 cells with or without herbal molecule treatments via proteomic approaches. The data were further analyzed by Ingenuity Pathway Analysis (IPA) and confirmed by quantifying mRNA associated with the estrogen-receptor signaling pathway and mitochondrial functions. We demonstrated that the expression of CYC1, TFAM, ATP5PB, mtATP6, mtDNA, and NRF-1 were decreased upon 12 h treatment, and subsequent ATP production was also significantly decreased at 24 h. These results identified a synergistic effect induced by combinational treatment with hesperidin and chlorogenic acid, which can regulate mitochondria and ATP production through the estrogen receptor pathway in MCF-7 cells. However, none of the treatments induced the generation of reactive oxygen species (ROS), suggesting that ROS likely plays no role in the observed pharmacological activities. Overall, our study sheds light on the adequacy of hesperidin and chlorogenic acid to serve as an adjunctive therapy when co-administrated with chemotherapy drugs in breast cancer patients.

## 1. Introduction

Breast cancer is the most prevalent cancer in women worldwide. In Taiwan, over 10 thousand people were diagnosed with breast cancer in 2013–2016, representing 25% of all new cancer cases in females [1]. While the incidence rate has been increasing slightly by an average of 0.28% annually, breast cancer has the highest incidence rate of all cancers. From 2011–2017, breast cancer was the fourth highest cause of cancer-related deaths, with 80% of patient having a 5-year relative survival rate [2,3]. Nevertheless, over 2000 breast cancer patients die annually, accounting for more than 10% of total deaths [4]. In line with Taiwan, breast cancer is also the fourth leading cause of cancer-related deaths in the US [5].

The positive outlook for a 5-year survival rate of breast cancer is largely owed to the existence of multiple effective therapeutic strategies, including surgery of operable tumors, neoadjuvant and adjuvant systemic therapy, and radiotherapy—all of which can be applied to control breast cancer depending on the patients’ condition and stage [6]. However, the inevitable undesired effects and possible recurrence remain primary concerns regarding the patient’s life quality and prognosis. As a result, combinational therapy has been considered a feasible approach to minimize toxicity and dosages while enhancing its efficacy and the resultant longevity of patients. For example, cyclin-dependent kinases 4/6 inhibitors, including palbociclib, ribociclib, and abemaciclib, have been approved for the treatment of patients with hormone receptor-positive and human epidermal growth factor receptor 2 (HER2)-negative advanced breast cancer [7].

Traditional herbal medicine is an ideal adjunctive candidate for combination with the aforementioned breast cancer therapies. It has been shown that medicinal herbs can alleviate adverse effects such as nausea, vomiting, and neuropathic pain caused by chemotherapies [8,9]. Moreover, herbal extracts, such as *Radix astragalus*, with immunomodulatory and anti-cancer effects have been evaluated for breast cancer therapy preclinically [10,11].

Hesperidin is a flavonoid that primarily exists in citrus fruits, such as grapefruit, lemon, or tangerines. Previous reports have indicated that the bioactivities of hesperidin include anti-inflammatory, anti-hypertensive, and anti-diabetic effects [12,13,14]. In addition, hesperidin has various anti-cancer activities including those against breast cancer [15]. Hesperidin can inhibit breast cancer by inducing cell cycle arrest and apoptosis [16]. Similarly, chlorogenic acid is a polyphenol isolated from *Phyllostachys edulis*, *Lonicera japonica* Thunb, or more abundantly from coffee [17]. The compound has also exhibited immunoregulatory and anti-angiogenic effects [18,19]. Of greater relevance, chlorogenic acid may suppress breast cancer cells via inducing protein kinase C-α to translocate to the cellular membrane and trigger apoptosis [20]. As a result, we selected these two herbal-derived compounds, hesperidin and chlorogenic acid, to investigate their efficacy, either singly or in combination, in MCF-7 breast cancer cells.

## 2. Materials and Methods

### 2.1. Cell Lines and Cell Culture

Human normal breast cell line, MCF-10A, was a generous gift from Dr. Huan-Tsung Chang at National Taiwan University, and the cancerous breast cell line, MCF-7, was directly purchased from the American Type Culture Collection (ATCC). MCF-7 and MCF-10A were cultured in Dulbecco’s Modified Eagle Medium (DMEM; ThermoFisher Scientific Inc., Waltham, WA, USA) and Minimum Essential Medium α (MEM α; ThermoFisher Scientific Inc.), respectively, supplemented with 10% fetal bovine serum (Biological Industries, Cromwell, CT, USA), 1× glutamine, and 1× antibiotics (Biowest, Nuaille, France) and incubated in a 37 °C humidified incubator with 5% CO_2_.

### 2.2. MTT Assay

MTT assay is a common method used to measure the metabolic changes of tetrazolium salts in viable cells to determine cellular viability and proliferation [21]. At 24 h pretreatment, MCF-7 and MCF-10A cells were seeded in 96-well plates at 8 × 10^3^ and 9 × 10^3^ cells per well, respectively. The cells were incubated further in the presence of herbal compounds at various concentrations. Subsequently, 20 μL of 5 mg/mL MTT solution (Sigma-Aldrich, St. Louis, MO, USA) in phosphate buffered saline (PBS) was added to each well containing DMEM. Following 4 h incubation, the supernatant was removed, and formazan was dissolved with dimethyl sulfide (DMSO; Sigma-Aldrich). The colorimetric changes were determined by a microplate reader at a wavelength of 490 nm.

### 2.3. Protein Extraction and Isobaric Labeling for Proteomics Analysis

MCF-7 treated with and without hesperidin and chlorogenic acid were collected and homogenized in lysis buffer (8 M urea in 50 mM triethyl ammonium bicarbonate (TEAB) buffer, pH 8) to extract proteins. Cell lysate was centrifuged at 16,000× *g* for 10 min at 4 °C, the supernatants were collected, and the protein concentration was determined by Bradford assay (Thermo Fisher Scientific Inc.) according to the manufacturer’s instructions. For reduction and alkylation of disulfide bonds in proteins, 100 μg of protein was treated with dithiothreitol (DTT) to a final concentration of 10 mM and then incubated at 55 °C for 30 min. Then, iodoacetamide (IAA) was added to a final concentration of 20 mM prior to incubation for 30 min at room temperature in the dark. Then, a second aliquot of DTT was added to quench unreacted IAA. Six volumes of pre-chilled (−20 °C) acetone was added to each protein sample and frozen at −20 °C for at least 4 h to precipitate proteins. The acetone-precipitated protein pellet was collected by centrifugation, resuspended with 50 mM triethylamonium bicarbonate (TEAB), and followed by the addition of trypsin (protein/trypsin ratio of 50:1) for digestion overnight at 37 °C. The peptide samples were labeled using a Tandem Mass Tag (TMT) six-plex isobaric label reagent set (Thermo Fisher Scientific Inc.) according to the manufacturer’s protocol. Each peptide solution was incubated for 1 h at room temperature and quenched for 15 min with 8 μL of 5% hydroxylamine solution. After labeling, the samples were desalted by Oasis HLB solid-phase extraction cartridges (Waters Corporation, Milford, MA, USA). Briefly, the cartridges were wet by acetonitrile followed by washing with 0.1% formic acid solution. The TMT-labeled peptide samples were loaded onto cartridges. After washing by 0.1% formic acid solution twice, peptides were eluted by 5% formic acid in 50% acetonitrile three times and dried by SpeedVac vacuum concentrators (SPD110, Thermo Fisher Scientific Inc.). In order to reduce the complexity and improve the protein identification and confidence level of quantification, the SpeedVac-drived TMT-labeled peptides were divided into 8 fractions by using a high-pH reversed-phase peptide fractionation kit (Thermo Fisher Scientific Inc.). Fractions were dried by vacuum concentrators and dissolved in 0.1% formic acid before Liquid Chromatography with tandem mass spectrometry (LC-MS/MS) analysis.

### 2.4. LC-MS/MS Analysis

MS data were acquired on an Orbitrap Fusion mass spectrometer (Thermo Fisher Scientific Inc.) equipped with an EASY-nLC 1200 system (Thermo Fisher Scientific Inc.), EASY-Spray HPLC column (75 μm I.D. × 150 mm, 3 μm, 100 Å), and ion source (Thermo Fisher Scientific Inc.). The chromatographic separation was performed using 0.1% formic acid in water as mobile phase A and 0.1% formic acid in 80% acetonitrile as mobile phase B operated at the flow rate of 300 nL × min^−1^. The LC gradient was employed from 2% buffer B at 2 min to 40% buffer B at 100 min. Electrospray voltage was maintained at 1.8 kV, and the capillary temperature was set at 275 °C. Full MS survey scans were executed in the mass range of m/z 320 to 1600 (AGC target at 5 × 10^5^) with lock mass, resolution of 120,000 (at m/z 200), and a maximum injection time of 50 ms. The MS/MS were run in top speed mode with 3 s cycles, while the dynamic exclusion duration was set to 60 s with a 10 ppm tolerance around the selected precursor and its isotopes. The precursor ion isolation was performed with mass selecting quadrupole, and the isolation window was set to m/z 2.0. Monoisotopic precursor ion selection was enabled and 1+ charge state ions were rejected for MS/MS. The MS/MS analyses were carried out with the high-energy collision dissociation (HCD) mode with the stepped collision energy of 31%, 35%, and 39%. The maximum injection time for spectra acquisition was 150 ms, and the automatic gain control (AGC) target values for MS/MS scans were set at 5 × 10^4^.

### 2.5. Proteomic Data Analysis

Acquired MS raw data were analyzed using MaxQuant (ver. 1.6.14) with UniProt human protein database (Taxonomy ID: 9606). For database searches, the precursor mass tolerance was set to 20 ppm for first searches and 10 ppm for the main database search. The fragment ion mass tolerance was set to 0.6 Da. Trypsin was chosen as the enzyme, and 2 missed cleavages were allowed. The carbamidomethylation of cysteine was defined as a fixed modification and the oxidation of methionine was defined as variable modification. Minimum peptide length was set to seven amino acids, and the minimum number of unique peptides was set to two. Maximum False discovery rate (FDR), calculated by employing a reverse database, were set to 5% for both peptides and proteins. Proteins identified as “reverse” and “only identified by site” were discarded from the list. The protein quantitation was determined by the reporter ion of 6-plex TMT in MS/MS. All quantifiable proteins were subjected to Orthogonal partial least squares discriminant analysis (OPLS-DA) (SIMCA, ver. 14) and bioinformatic pathway analysis.

### 2.6. ROS Detection Cell-Based Assay

The production of reactive oxygen species (ROS) is a critical indication of oxidative pressure [22]. MCF-7 cells were seeded in 96-well plates at 2 × 10^4^ cells per well for 24 h followed by herbal compound treatments for 12 h. After removing culture medium, fresh medium with 10 µM 2′-7′dichlorofluorescin diacetate (DCFH-DA) was added to each well, including positive control wells which were subject to 30 min incubation with 1 mM H_2_O_2_. DCFH-DA medium was removed and washed off twice with PBS. Finally, wells with 100 μL of PBS were either measured with a microplate reader or examined under a fluorescence microscope (Olympus IX71, Olympus Life Science, Tokyo, Japan).

### 2.7. ATP Assay

MCF-7 cells were seeded at 10^6^ cells/well in 6-well plates and incubated at 37 °C for 24 h followed by the addition of herbal compounds for another 12 h. Then, cells were trypisinized and collected in tubes and washed twice with PBS. Cell pellets were sonicated to obtain protein extract. After quantifying protein concentrations by the bicinichoninic acid (BCA) method (#23225 Thermo Scientific pierce BCA protein assay kit, Thermo Fisher Scientific Inc., Waltham, MA, USA) and boiling for 10 min to deactivate ATPase, total proteins were centrifuged at 20,000× *g* for 5 min. Then, 5 µL of each protein sample was mixed with 10 µL dH_2_O and 35 µL of 4 mM Ethylenediaminetetraacetic acid (EDTA), and 5 µL of the mixture was combined with 100 µL of ATP Assay Solution, and the mixtures were immediately measured for relative light unit (RLU) of luminescence as a quantification of ATP concentration in each sample according to the ATP assay kit protocol (Biomedical Research Service Center, Buffalo, NY, USA).

### 2.8. Reverse-Transcription PCR and Quantitative PCR

The total mRNAs of MCF-7 cells treated with herbal compounds for 6 h or 12 h were quantified by Nanodrop (ThermoFisher Scientific). One µg of mRNA was mixed with 1 µL random primers in a tube and run on a PCR device at 65 °C for 5 min and set on ice. To generate cDNA, 4 µL of 5× Reaction Buffer, 2 µL of 10 mM of dNTP Mix, 1 µL of RiboLock RNase inhibitor, and 1 µL of RevertAidM-MuLV TR were added to the tubes, and the reactions were run in a PCR device (ASTEC) at 42 °C for 60 min and 70 °C for 5 min. In triplicate, 1 µL of cDNA was added to 10 µL of 2× SYBR master mix and 0.5 µL of 10 µM forward and reverse primer (Table 1), and reactions were performed with a quantitative PCR (Agilent Technoogies Strategene Mx3000P). The fold-changes of mRNA were defined as 2^ΔΔ^^Ct^, which were calculated using the following equations:

ΔCt_sample_ = Ct_target gene_ − Ct_internal control gene_ ΔCt_conrol_ = Ct_target gene_ − Ct_internal control gene_ ΔΔCt = ΔCt_sample_ − ΔCt_conrol_.

### 2.9. Statistical Analysis

All data in the study were presented as mean ± S.D. In determing significance, data between any two groups were compared by one-way Analysis of Variance (ANOVA) unless indicated otherwise via software GraphPad Prism v8.4 software (GraphPad Software; San Diego, CA, USA). Statistical significance was defined as a *p*-value under 0.05.

## 3. Results

### 3.1. Effects of Hesperidin and Chlorogenic Acid on Breast Cancer Cells

**Figure 1:**
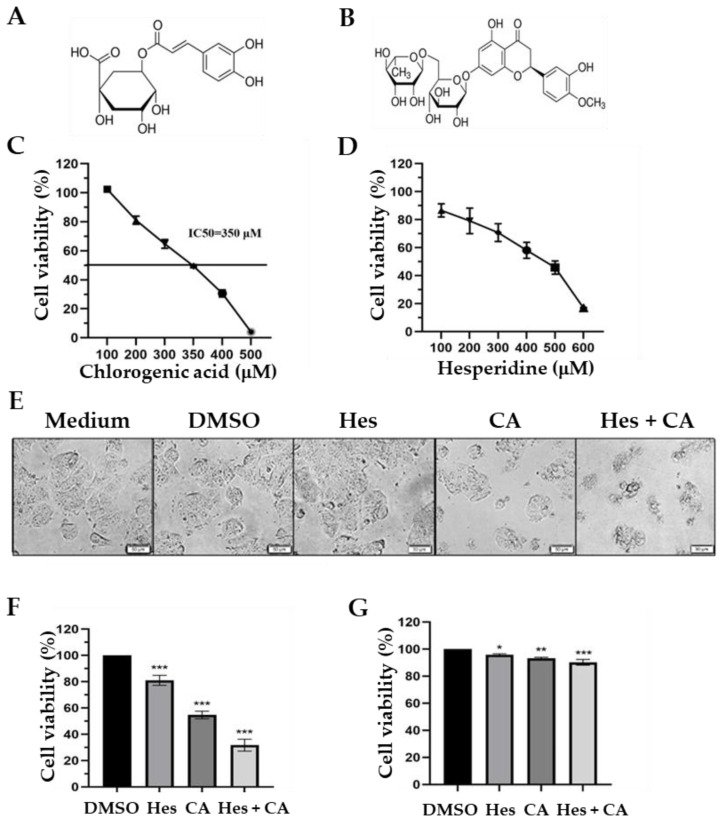
Cytotoxic effects of hesperidin and chlorogenic acid on breast cancer cells. The molecular structure of chlorogenic acid (**A**) and hesperidin (**B**), and the cytotoxicity to MCF-7 cells caused by chlorogenic acid (**C**) and hesperidin (**D**) determined by MTT assays after 72 h treatment at indicated concentrations. Cell morphology (**E**) and cell viability of MCF-7 (**F**) and MCF-10A (**G**) after treating with 100 μM hesperidin and/or 350 μM chlorogenic acid. Data presented as mean ± SD. * *p* < 0.05; ** *p* < 0.01; *** *p* < 0.001.

Firstly, we evaluated the anti-cancer activity of chlorogenic acid (Figure 1A) and hesperidin (Figure 1B) on MCF-7 cells. Various concentrations (100–600 µM) of hesperidin or chlorogenic acid were added to MCF-7 cells for 72 h followed by the determination of cell viability via MTT assay. The results indicate that chlorogenic acid exhibited a dose-dependent growth inhibition on MCF-7 cells, demonstrating a median inhibitory concentration (IC_50_) of 350 µM (Figure 1C). However, despite similar cytotoxicity at 100–600 µM, the IC_50_ of hesperidin was not determinable due to hesperidin crystallizing at higher concentrations (Figure 1D and Supplementary Figure S1).

To investigate whether any synergistic effect exists while applying both hesperidin and chlorogenic acid, we treated MCF-7 cells separately with 100 µM hesperidin, 350 µM chlorogenic acid, or both and determined the cell morphology and cell viability by light microscopy and MTT assays, respectively. As shown in Figure 1E, the cellular morphology of MCF-7 cells was more aggravated after combinational treatment of hesperidin and chlorogenic acid than during either treatment singly. MTT assay results reveal that 100 µM hesperidin and 350 µM chlorogenic acid caused around 20% and 50% cell death, whereas 30% cell viability was found in the combinational treatment (Figure 1F). Therefore, the combination index (CI) of combinational treatment with hesperidin and chlorogenic acid determined by the Chou–Talalay method was 0.76 (Table 2), indicating that the drug combination acts synergistically [11]. Notably, the cytotoxicity induced by hesperidin and chlorogenic acid was more significant in cancerous cells (MCF-7) than normal breast cells (MCF-10A) after 24 h treatment (Figure 1G), suggesting that the cytotoxicity could be relatively tumor cell specific and less harmful to normal cells.

### 3.2. Identification of Pathways Affected by Hesperidin and Chlorogenic Acid

**Figure 2:**
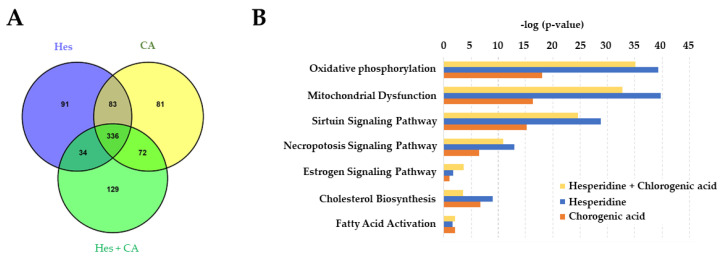
Proteomic analyses revealed possible pathways in MCF-7 cells. (**A**) Data obtained from mass spectrometry after 12 h treatment of hesperidin (100 μM) and chlorogenic acid (350 μM) was analyzed in MaxQuant by using the UniProt human protein database and presented by Venn diagram to show the numbers of total detectable proteins. (**B**) Protein expression changed by 20% (Hes), 30% (CA), and 40% (Hes + CA) were selected and aligned via Ingenuity Pathway Analysis for the identification of top disease biofunctions.

In order to pin down the underlying pathways modulated by hesperidin and chlorogenic acid in MCF-7, leading to the anti-cancer activity, we screened the changes in protein expression 12 h after treatment by mass spectrometry (flow chart shown in Supplementary Figure S1). The isobaric tag TMT was used to label tryptic peptides to improve the accuracy of protein quantitation. MS data were analyzed in MaxQuant by using the UniProt human protein database, and 5183 associated proteins were quantifiable out of a total of 5355 identified proteins. Compared to the experimental control sample (DMSO treatment), it was found that 544 (hesperidin-treated), 572 (chlorogenic acid-treated), and 571 (combination) proteins showed expression changes of 20%. Among them, 336 proteins were found to be involved in all three different treatment conditions, as shown in the Venn diagram in Figure 2A. Additionally, these proteins were subject to Ingenuity Pathway Analysis (IPA) to clarify the regulatory pathways and biological functions affected by the treatment of hesperidin and chlorogenic acid treatment. IPA results indicate that multiple ingenuity canonical pathways were affected such as estrogen receptor signaling, which was mentioned in the previous studies (Figure 2B). Furthermore, IPA analysis showed that hesperidin and chlorogenic acid have significant effects on oxidative phosphorylation, mitochondrial dysfunction, and the sirtuin signaling pathway. The biological function analysis of IPA shows that the synthesis of ATP and synthesis of lipids functions were both downregulated in the presence of hesperidin and chlorogenic acid.

### 3.3. Estrogen-Receptor Pathways Were Modulated by Hesperidin and Chlorogenic Acid

**Figure 3:**
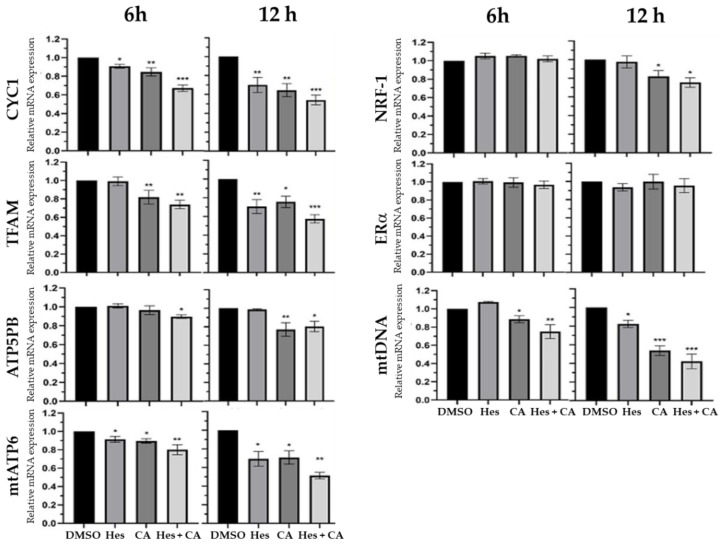
mRNA expressions in MCF-7 cells after treatments. Seven estrogen-receptor pathway-associated genes were measured for fold changes of mRNA expressional levels against internal reference gene, β-actin, at 6 and 12 h of 100 μM hesperidin and/or 350 μM chlorogenic acid treatments. Culture medium containing 0.1% DMSO was used for vehicle control. Data presented as mean ± SD. * *p* < 0.05; ** *p* < 0.01; *** *p* < 0.001.

Considering that the estrogen-receptor pathway influences the transcription, translation, and synthesis of ATP in mitochondria [23], and to confirm the findings obtained from proteomics, we examined seven upstream and downstream genes, which are associated with the estrogen-receptor pathway following hesperidin and chlorogenic acid treatment for 6 and 12 h. These genes included nuclear respiratory factor 1 (NRF-1), estrogen receptor α (ERα), mitochondrial transcription factor A (TFAM), cytochrome c (CYC1), ATP synthase subunit B (ATP5PB), mitochondrial membrane ATP synthase (mtATP6), and mitochondrial DNA (mtDNA). Results showed that 6 h combinational treatment significantly reduced the gene expressions of CYC, TFAM, mtATP6, ATP5PB, and mtDNA—but not NRF-1 and ER*α*—by 10–30% compared to DMSO control (Figure 3). By 12 h, the synergistic effects further reduced the aforementioned gene expressions by 40–60% with slight reduction of NRF-1 but no change in ER*α*, suggesting the hesperidin and chlorogenic acid treatment significantly stymied the downstream genes in the estrogen-receptor pathway.

### 3.4. Combinational Treatments Reduced ATP Synthesis but Did Not Induce ROS Production

**Figure 4:**
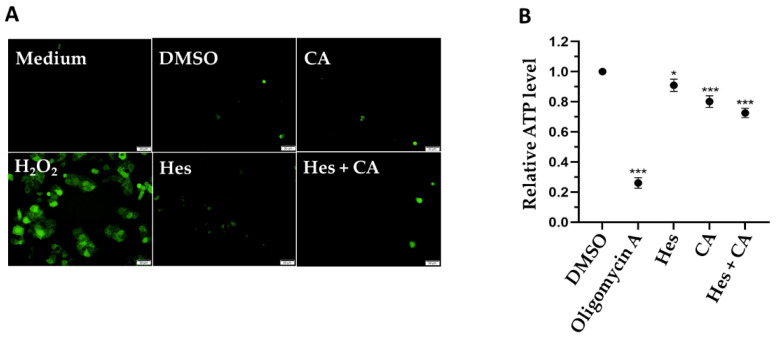
Potential acting mechanism exerted by selected medicinal herbal compounds. (**A**) ROS productions and (**B**) total ATP concentrations in MCF-7 cells after treatments (100 μM of Hes; 350 μM of CA) at 24 h were detected via fluorescent DCFH-DA and observed under a microscope. Hydrogen peroxide (H_2_O_2_) was used for positive control to induce ROS, whereas medium and DMSO control were normal and vehicle controls, respectively. Data presented as mean ± SD. * *p* < 0.05; *** *p* < 0.001.

Previous studies indicate that the induction of ROS might be a mechanism for hesperidin and chlorogenic acid that inhibits the proliferation of cancer cells [24,25]. Therefore, we detected the ROS production via the addition of fluorescence-emitting DCFH-DA and examined the fluorescent signals utilizing microscopy. Interestingly, while the hydrogen peroxide positive control induced strong ROS production, there was no significant ROS in MCF-7 cells treated by either single or combinational treatment with both hesperidin and chlorogenic acid (Figure 4A).

Since estrogen-receptor-related genes were affected by hesperidin and chlorogenic acid treatments, we considered if the treatments suppressed the physiological functions of mitochondria and ultimately reduced ATP synthesis. To this end, we measured the intracellular ATP concentrations. Our results clearly show that compared to the DMSO vehicle control and positive control in cells treated with the ATPase inhibitor, oligomycin A, for 4 h, hesperidin and chlorogenic acid significantly reduced the ATP concentrations by 10% and 20%, respectively. Furthermore, the combinational treatment synergistically reduced ATP levels by 28% (Figure 4B), revealing that the inhibition of ATP synthesis could be one of the profound underlying mechanisms exerted by hesperidin and chlorogenic acid to suppress breast cancer cells.

## 4. Discussion

Medicinal herbal compounds have served as adjunctive therapies in different diseases preclinically and clinically. For example, ginseng is known for its activity against prostate, melanoma, and colon cancers. It has also been evaluated to work in combination with cisplatin to treat non-small-cell lung cancer (NSCLC) patients in clinical trials, as ginseng is able to enhance the cytotoxicity of multiple chemotherapy drugs [26]. Regarding breast cancer, hesperidin was shown to increase the cytotoxicity of tamoxifen, a selective estrogen receptor modulator, and imatinib mesylate, a platelet-derived growth factor (PDGF) receptor inhibitor, when treating breast cancer cells [27,28]. In addition, combination with hesperidin could potentially prevent the resistance of MCF-7 to doxorubicin treatment in breast cancers [29]. Even a single usage of hesperidin was shown to moderately reduce the colony formation of MCF-7 cells [30]. In accordance with previous reports regarding hesperidin, our findings demonstrated that hesperidin along with chlorogenic acid prominently enhanced the toxicity against MCF-7, but was less harmful to normal breast cells, MCF-10A, indicating the feasibility of such a combinational treatment (Figure 1).

Parallelly, chlorogenic acid was found to reduce breast tumor sizes in rats and the 4T1 mouse model by disturbing the tumor microenvironment via the suppression of vascular endothelial-derived growth factor (VEGF, for angiogenesis), cluster of differentiation 34 (CD34, a common marker for identifying hematopoietic stem cells (HSCs)), Transforming growth factor (TGF-β, a key mediator of angiogenesis) expression, and by reducing the nuclear translocation of NF-κB, respectively [31,32]. Consequently, we inferred that the synergistic effect in suppressing breast cancer cells that we observed in this study could be a result of the distinct mechanisms of action exerted by hesperidin and chlorogenic acid. Our study is not only in concurrence with previous research but also provides implications for combinational use of medicinal herbal compounds.

Despite the lack of extensive studies on chlorogenic acid, Rosendahl et al. reported that caffeic acid could reduce the progression of breast cancer cells alongside tamoxifen treatment [33]. Our findings demonstrate that chlorogenic acid also potentially modulates the estrogen-receptor pathway to achieve a similar anti-cancer property as hesperidin (Figure 3). Therefore, it is reasonable to assume that a synergistic benefit may exist in combined therapy with an estrogen-receptor-targeting chemotherapy drug.

**Figure 5:**
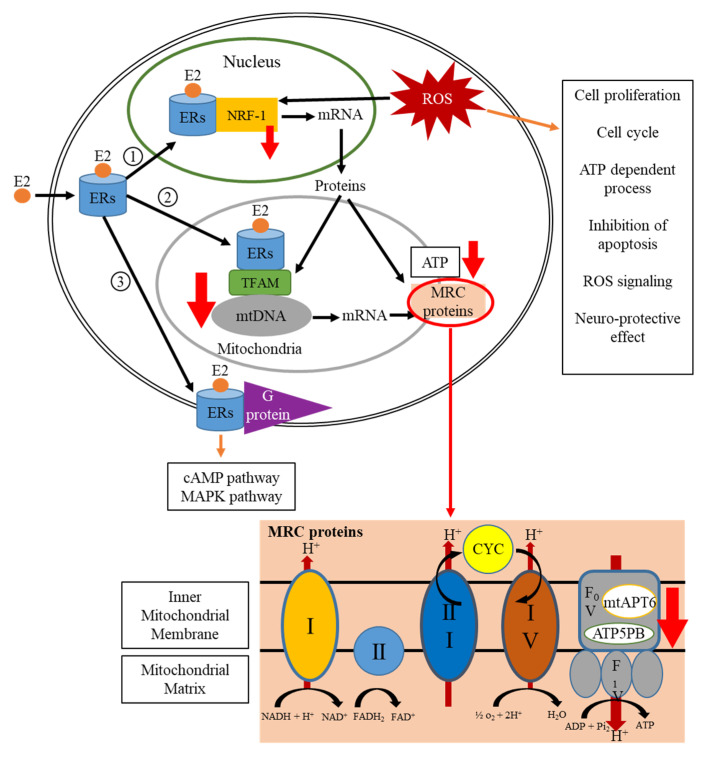
The scheme of the estrogen-receptor pathway associated with treatments. Red arrows designated for gene expressional changes.

Modulation of estrogen-receptor associated signaling appears to be one of the major pathways exerted by hesperidin and chlorogenic acid acting in concert. Data obtained from the quantitation of related RNA expressions indicate that the first six-hour treatment reduced the expression of downstream genes, including TFAM, NRF-1, and mtDNA as well as ATP-synthetic-related genes, ATP5PB and mtATP6 (Figure 5). However, upstream genes, such as ERα, remained relatively unaffected regardless of the selected time points, suggesting that hesperidin and chlorogenic acid act through ERα at the earlier time point of 6 h and more directly influence the physiological functions and activity of mitochondria as corroborated by IPA results (Figure 2B). In addition to the mitochondrial dysfunction and oxidative phosphorylation pathways, our proteomic data also suggested that necroptosis and apoptosis initiated by hesperidin or chlorogenic acid, for example, could also be involved in anti-cancer bioactivity [34,35]. Moreover, the insulin secretion pathway, including insulin growth factor 1 (IGF-1) and receptor (INSR) gene expressions, was shown to be downregulated by Hes and CA (Table 3). It is clinically implicated that hyperinsulinemia or diabetes could be one of the risk factors inducing breast cancers and that the active insulin pathway could also facilitate the progression and metastasis of breast cancer via providing excessive nutrients and pro-survival signals [36,37]. Nevertheless, the detailed mechanisms underlying how Hes and CA modulate the insulin pathway in a breast cancer cell are worthy of investigation. 

Notably, among various pathways that were downregulated in breast cancer cells, we observed that the sirtuin signaling pathway was upregulated upon combinational treatment (Table 3). This result was in accordance with the previous evidence, supporting the notion that SIRT1 expression could be highly associated with the BRCA1 mutation and the potential role of SIRT1 as a tumor suppressor in breast cancers [38,39]. However, the dual properties of sirtuin proteins in breast cancers are debatable and require further investigation in the future [40].

Previous studies showed that both hesperidin and chlorogenic acid could induce ROS production in human gall bladder carcinoma and colon cancer cells, respectively [24,25]. However, we did not observe any increase in ROS in this study (Figure 4A). In addition, the downregulation of the NRF-1 gene was moderately detected only after 12 h of combinational treatment (Figure 3). As a result, we reasoned that the synergistic effects of hesperidin and chlorogenic acid might not be due to ROS-related genes and only partially inhibit ATP synthesis via NRF-1.

Targeted therapy has been applied in breast cancer treatments and provides a better post-diagnosis in terms of survival outcomes [41,42]. Despite this, targeted therapy necessitates the development of methods to precisely deliver anti-cancer compounds. Among numerous attempts to develop such a capacity, nanoencapsulation has been extensively exploited as a novel strategy to deliver anti-cancer drugs particularly for flavonoid phytochemicals due to their poor bioavailability [43,44]. The use of chitosan, rebaudioside A, and directly loading onto gold nanoparticles all have been shown to be feasible approaches and profoundly enhance the anti-cancer activity of chlorogenic or hesperidin [45,46,47,48]. With this concept in mind, our next endeavor is to encapsulate our herbal compounds with chitosan or nanolize chlorogenic acid and hesperidin via dry-heating treatment [49] to assess the delivery efficacy and therapeutic improvement in a murine model.

## 5. Conclusions

Our study demonstrates that a combinational use of hesperidin and chlorogenic acid profoundly suppressed breast cancer cells, while limiting damage to normal breast cells. Moreover, proteomic and mRNA analyses supported evidence that downstream estrogen-receptor genes were downregulated upon combinational treatment, ultimately reducing ATP production in the mitochondria of a cancerous cell. Hence, hesperidin and chlorogenic acid could thereby be suitable for adjunctive therapy when applying chemotherapy.

## Figures and Tables

**Table 1:** Primer pairs used in the quantification of mRNA.

Gene Name	Forward Primer (5′–3′)	Reverse Primer (5′–3′)
**CYC1**	5′-CCAGATAGCCAAGGATGTGTGC-3′	5′-GACTGACCACTTGTGCCGCTTT-3′
**TFAM**	5′-AGCTCAGAACCCAGATGC-3′	5′-CCACTCCGCCCTATAAGC-3′
**ATP5PB**	5′-TCACAGGGACGCTAAGATTGC-3′	5′-CCTTGTTGCCTGCAATACCC-3′
**mtDNA**	5′-ACACCCTCCTAGCCTTACTAC-3′	5′-GATATAGGGTCGAAGCCGC-3′
**mt-ATP6**	5′-GAAGCGCCACCCTAGCAATA-3′	5′-GCTTGGATTAAGGCGACAGC-3′
**NRF-1**	5′-CCACGTTACAGGGAGGTGAG-3′	5′-TGTAGCTCCCTGCTGCATCT-3′
**ERα**	5′-ACTGCAGGATGAGCTGG-3′	5′-TGCACAGAGTCTGAATTGG-3′

**Table 2:** CI values of combinational treatment of hesperidin and chlorogenic acid.

Combinational Treatment			
Hes Dose (μM)	CA Dose (μM)	Cell Death (%)	CI Value
100	350	68.22	0.76

**Table 3:** IPA results of canonical pathways influenced by Hes and CA.

Ingenuity Canonical Pathways	−log(*p*-Value)	Ratio	Z-Score
Oxidative Phosphorylation	35.1	0.284	−5.568
Sirtuin Signaling Pathway	24.6	0.117	2.138
Necroptosis Signaling Pathway	10.9	0.102	−2.5
Induction of Apoptosis by HIV1	4.32	0.0984	−2.449
Estrogen Receptor Signaling	3.64	0.0366	−2.887
Insulin Secretion Signaling Pathway	2.9	0.037	−1.667
Systemic Lupus Erythematosus in B Cell Signaling Pathway	0.47	0.0145	0
Senescence Pathway	0.47	0.0145	−1
Hepatic Fibrosis Signaling Pathway	0.446	0.0136	−0.447
Cardiac Hypertrophy Signaling	0.377	0.0123	−0.447
Protein Kinase A Signaling	0.374	0.0125	0
Synaptogenesis Signaling Pathway	0.369	0.0128	−1
Insulin Secretion Signaling Pathway	2.9	0.037	−1.667

## Data Availability

The data supporting the findings of this study are available in the article.

## Supplementary Materials

The following are available online at www.mdpi.com/article/10.3390/life11090950/s1, Figure S1: Representative photos showing crystallization of hesperidin at concentration higher than 100 μM with or without chlorogenic acid, Figure S2: Flow chart of sample preparation and analysis of mass-spectrometry.
