# Supplementary material for: Hesperidin and Chlorogenic Acid Synergistically Inhibit the Growth of Breast Cancer Cells via Estrogen Receptor/Mitochondrial Pathway
Source: Life (Basel). 2021 Sep 10;11(9):950. doi: 10.3390/life11090950 (PMC8467139; doi:10.3390/life11090950)
Supplement: Supplementary file 1 [file life-11-00950-s001.zip › life-1351399-supplementary.pdf]

life

Supplementary Materials

Hesperidin and Chlorogenic Acid Synergistically Inhibit the Growth of Breast Cancer Cells via Estrogen Receptor/Mitochondrial Pathway

Pang-Hung Hsu [1,2,3,†], Wei-Hsuan Chen [1,†], Chen JuanLu [4,†], Shu-Chen Hsieh [4], Shih-Chao Lin [5], Ru-Tsun Mai [6] and Shiow-Yi Chen [1,*]

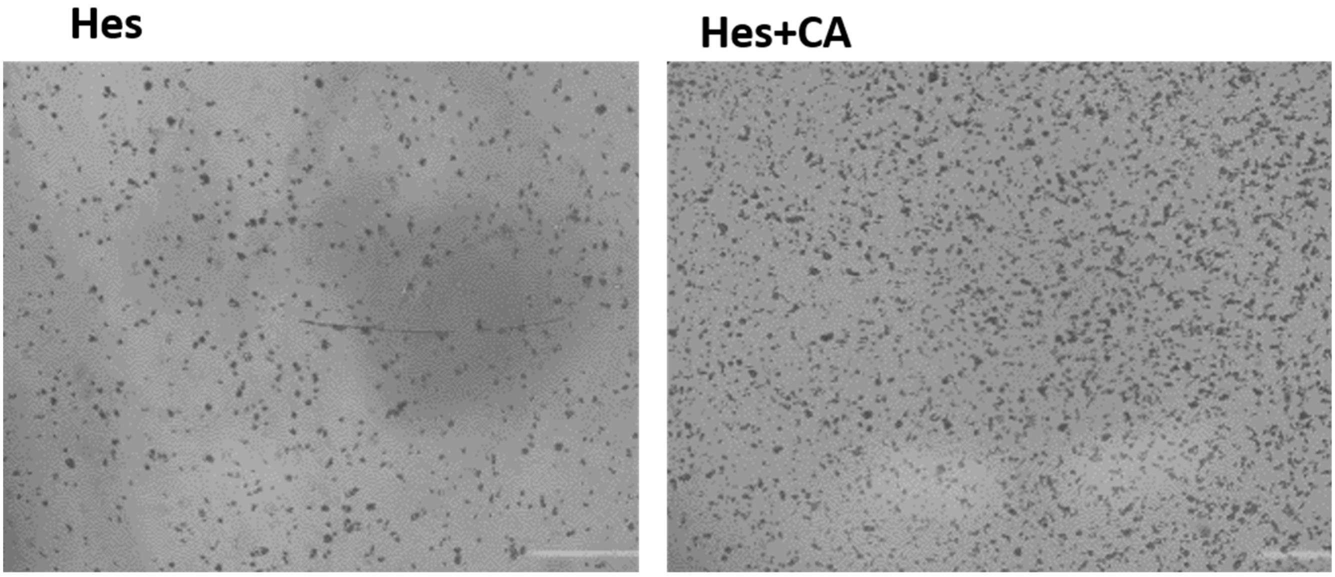

Figure S1. Representative photos showing crystallization of hesperidin at concentration higher than 100 μM with or without chlorogenic acid.

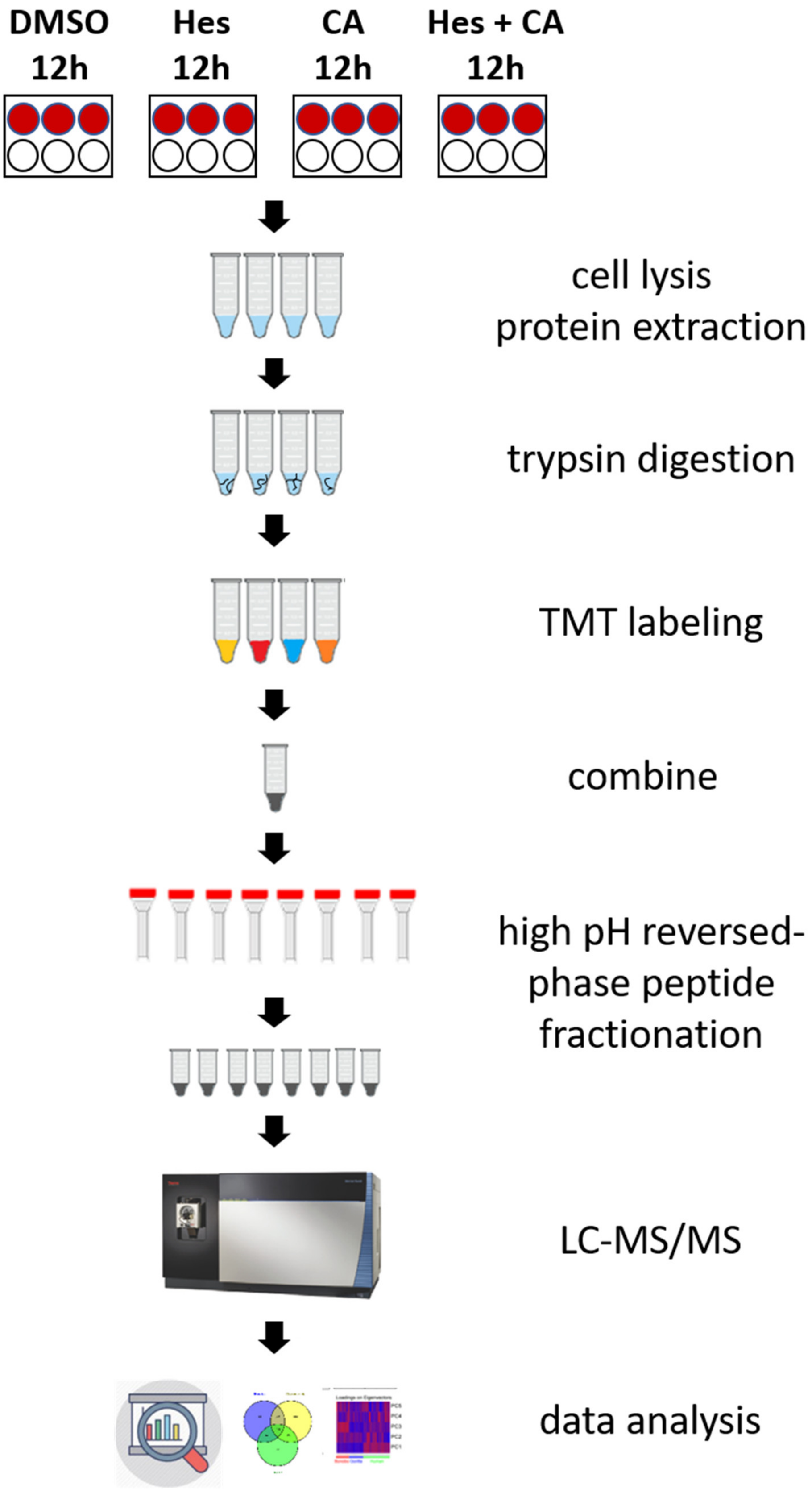

Figure S2. Flow chart of sample preparation and analysis of mass-spectrometry.
